# High expression levels of TLR9 and PD-L1 indicates a poor prognosis in patients with angioimmunoblastic T-cell lymphoma: a retrospective study of 88 cases in a single center

**DOI:** 10.7150/jca.37033

**Published:** 2020-01-01

**Authors:** Jingrong Qian, Hongxue Meng, Bowen Lv, Jie Wang, Yingying Lu, Liju Su, Shu Zhao, Wenhui Li

**Affiliations:** 1 Department of Clinical Laboratory, Harbin Medical University Cancer Hospital, Harbin, Heilongjiang 150081, P. R. China.; 2 Department of Pathology, Harbin Medical University Cancer Hospital, Harbin, Heilongjiang 150081, P. R. China.; 3 Department of Clinical Laboratory, The First Hospital of Harbin, Harbin, Heilongjiang 150010, P. R. China.; 4 Department of Medical Oncology, Harbin Medical University Cancer Hospital, Harbin, Heilongjiang 150081, P. R. China.

**Keywords:** TLR9, PD-L1, angioimmunoblastic T-cell lymphoma, prognosis

## Abstract

**Background:** The role of TLR9 expressed by tumor cells in evading immune surveillance was confirmed. PD-L1 expression in tumor cells plays a key role in tumor immune escape, which is associated with poor prognosis. However, the clinical relevance of TLR9 and PD-L1 expression in angioimmunoblastic T-cell lymphoma (AITL) has not been evaluated.

**Materials and methods:** In this study, we identified differentially expressed genes in AITL samples by bioinformatic analysis, and we first examined TLR9 and PD-L1 expression by immunohistochemical staining in patients with AITL and compared these data with clinical features and survival time.

**Results:** It was found that the expression of PD-L1 and multiple TLRs was higher in AITL than normal T-cell samples, and TLR9 and PD-L1 expression displayed complex interactions by bioinformatic analysis. The rates of TLR9 and PD-L1 high expression were 69% and 50%, respectively. High expression of either TLR9 or PD-L1 indicated a poor survival rate for patients with AITL. Multivariate analysis further confirmed that high expression levels of TLR9 and PD-L1 were unfavorable prognostic factors for AITL. We further found inferior overall survival in AITL with clinical features of ECOG status ≥ 2, advanced-stage, elevated serum LDH levels, elevated serum β_2_-MG levels, and high IPI score.

**Conclusion**: TLR9 and PD-L1 expression may be a novel predictor of prognosis for patients with AITL and may serve as potential therapeutic strategy.

## Introduction

Angioimmunoblastic T-cell lymphoma (AITL) is an age-related aggressive lymphoma and belongs to the nodal T-cell lymphoma with T follicular helper (Tfh) phenotype 1. It displays a unique morphologic appearance and is biologically complex 2. Despite treatment with various therapies, the survival rate of AITL remains bleak with a 5-year overall survival (OS) rate of 30% 3. Data on the prognostic factors of AITL are limited, and innovative therapeutic targets remain to be determined.

Programmed cell death protein-1 (PD-1) is a member of the CD28/CTLA-4 family of co-stimulatory receptors and conveys inhibitory signals to T cells 4. Programmed death ligand-1 (PD-L1) is a ligand for PD-1 and is reported to be expressed on tumor cells. The high expression of PD-L1 is associated with poor clinical prognosis in some solid tumors 5,6 and in some subtypes of T- and B-cell lymphomas, including adult T-cell leukemia/lymphoma and diffuse large B-cell lymphoma 7,8. However, the clinical prognostic implications of PD-1 and PD-L1 expression in AITL are unknown.

Toll-like receptor9 (TLR9), a member of the toll-like receptor family of pattern recognition receptors, binds unmethylated CpG DNA motifs present in microbial nucleic acids and activates the immune response, and is currently under investigation as an adjuvant in anti-PD-1 therapy 9,10. Recent studies reported that TLR9 is also expressed in some types of tumor cells, including oral cancer, breast cancer, glioma and pancreatic cancer cells. Some researchers suggested that high expression of TLR9 is associated with tumor growth and migration, and it has been recognized as a new prognostic biomarker in cancer 11,12. To date, there has been limited information about the expression and clinical significance of TLR9 in AITL.

In this study, we identified differentially expressed genes in AITL samples, and using immunohistochemical staining, we analyzed TLR9 and PD-L1 expression in patients with AITL. And we further evaluated the relationship between TLR9 and PD-L1 expression, and compared these data with clinical features and survival time, which may disclose clinical significance of TLR9 and PD-L1 expression in AITL.

## Material and methods

### Genes expression profiles

Gene expression profiles GSE6338 were downloaded from the Gene Expression Omnibus database (GEO; Affymetrix GPL570 platform, Affymetrix Human Genome U133 Plus 2.0 Array; https://www.ncbi.nlm.nih.gov/geo/) database. We compared gene expression profiles between 5 AITL samples (GSM146170-GSM146174) and 5 normal T-cell samples (GSM146182-GSM146186). Those data were analyzed by the R Statistical Package (R version 3.4.3) to identified differentially expressed genes (DEGs). Each sample has significantly different gene expression profiles. Fold change (FC) > 2, adjusted *P*-value (adj. P) <0.001, and false discovery rate (FDR) < 0.01 were considered statistically significant.

### Functional bioinformatics microarray analysis

To analyze the function of DEGs, biological analyses were performed using The Database for Annotation, Visualization and Integrated Discovery online database (DAVID, version 6.8; https://david.ncifcrf.gov/). In this online database, we input DEGs into the Gene Ontology (GO) and Kyoto Encyclopedia of Genes and Genomes (KEGG) databases to annotate genes and analyze potential functions. The protein-protein interaction (PPI) network was predicted using the Search Tool for the Retrieval of Interacting Genes online database (STRING, version 10.5; https://string-db.org/), and was constructed using Cytoscape (version 3.6.1). Interactions with a combined score > 0.4 were considered statistically significant.

### Patient information

A total of 88 patients diagnosed with AITL at Harbin Medical University Cancer Hospital from January 2008 to June 2018 were enrolled. The diagnosis of AITL was based on criteria established by the World Health Organization criteria (WHO 2016) 1. Risk stratification was assigned according to international prognostic index (IPI) scores (low risk: 0-1; low-intermediate risk: 2; high-intermediate risk: 3; high risk: 4-5). The responses of treatments were defined according to the International Working Group response criteria (published in 2007) 13 or revised response criteria (published as the Lugano classification, 2014) 14.

### Immunohistochemistry staining

Immunohistochemical staining was performed on each of paraffin-embedded biopsy specimens obtained from 88 patients with AITL, a 3-μm thick formalin-fixed paraffin-embedded section was submitted. Immunohistochemical staining was performed, as previously described 15. The primary antibodies used for staining include: anti-TLR9 (ab134368, 1:100; Abcam, Cambridge, UK), anti-PD-L1 (ab205921, 1:100; Abcam, Cambridge, UK), and anti-PD-1 (ab52587, 1:100; Abcam, Cambridge, UK).

### Determination of TLR9 and PD-L1

TLR9 and PD-L1 expression was determined by immunohistochemical staining of adjacent tissue sections from the same biopsy specimen. The IHC scoring used for TLR9 and PD-L1 was evaluated independently by two experienced pathologists. The IHC score was acquired by a semi-quantitative method that takes staining intensity and cell proportion into account, and final IHC scores were consensus-based. For TLR9, staining intensity was divided into four categories according to the color of the immune reaction: negative, 0; light brown, 1; brown, 2; and dark brown, 3. The proportion of positively stained cells was determined and scored as: ≤ 5%, 0; 6-25%, 1; 26-50%, 2; 51-75%, 3; and 76-100%, 4. The expression level of TLR9 was obtained by multiplying the intensity and proportion score. And a final score of 0-2 was considered as negative expression (-, noted as 0); 3-4: weak positive expression (+, noted as 1); 6-8: moderate positive expression (++, noted as 2); and 9-12 was strong positive expression (+++, noted as 3) 15. For PD-L1, based on previous studies, the samples with stained tumor cells < 5% was defined as negative (-, scored as 0). For ≥ 5% positive stained samples, the expression level was further classified into weak positive (+, noted as 1), moderate positive (++, noted as 2), and strong positive expression (+++, noted as 3) according to staining intensity from light brown to brown and dark brown, correspondingly 16. Samples with negative and weak positive expression were defined as low expression, and samples with moderate and strong positive expression were defined as high expression for TLR9 and PD-L1.

### Statistical analysis

Clinicopathological characteristics of the patients were compared using the chi-square test or Fisher's two-sided exact test. The Kaplan-Meier method was used to estimate the overall survival (OS) and progression-free survival (PFS), and the Log-rank test was performed to determine significant differences. Univariate and multivariate Cox proportional regression models were used to evaluate the proposed prognostic factors. *P*-values < 0.05 were considered statistically significant. Data were analyzed using IBM SPSS software (version 22.0).

## Results

### Differentially expressed genes in AITL

**Figure 1:**
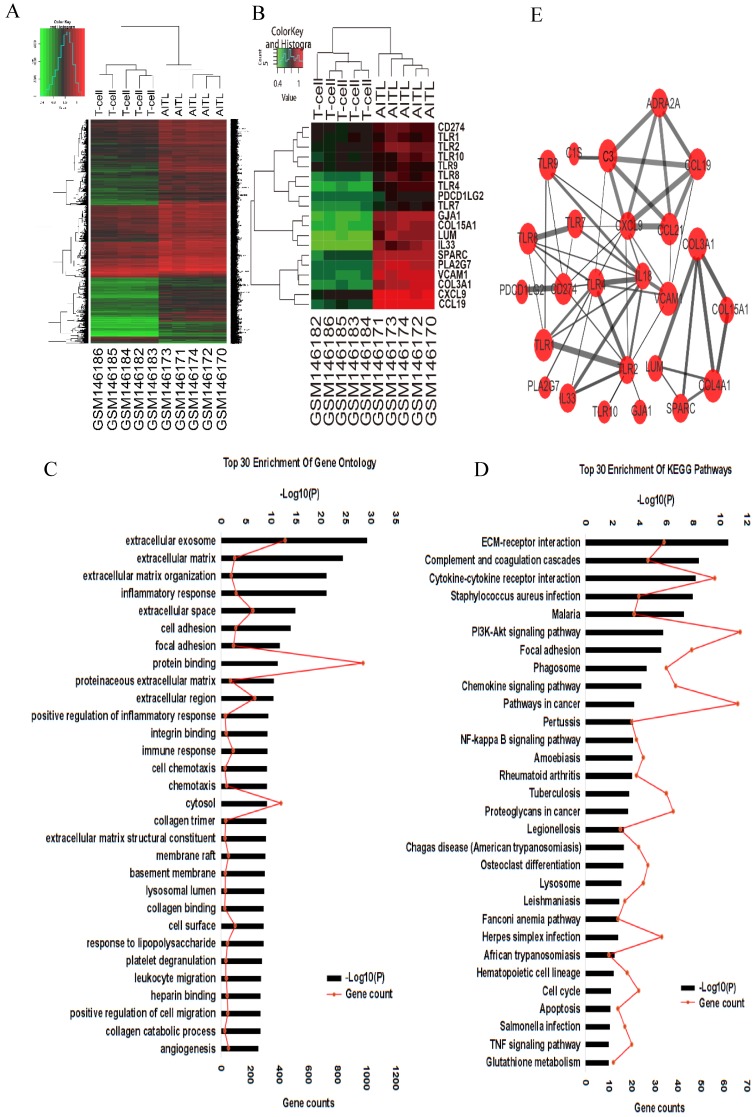
Gene expression profiling analysis in AITL. **(A)** Hierarchical clustering analysis of 5 AITL samples and 5 normal T cell samples was constructed using the R Statistical Package. A total of 10 samples were clustered according to the expression of 4,439 DEGs. **(B)** A total of 10 samples were clustered according to the expression of 19 DEGs related to immunological functions. **(C-D)** Top 30 enrichment GO terms and KEGG pathways for DEGs.** (E)** PPI network of DEGs was constructed.

We compared gene expression profiles between 5 AITL samples and 5 normal T cell samples by bioinformatic analysis. A total of 4,439 DEGs in AITL samples were identified, of which 4,182 were upregulated and 257 were downregulated (absolute FC > 2, FDR < 0.01, *P* < 0.001; Fig. 1A). We assessed the predictive functions of DEGs by GO enrichment analysis, several biological processes such as extracellular exosome, inflammatory response, immune response, cell chemotaxis, cell migration, and angiogenesis were enriched (Fig. 1C). Based on GO enrichment analysis, we suggested some key DEGs are enriched in inflammatory response (IL18, TNF/TNFRSF, TLR9, CXCL10, S1PR3, NLRC4, MYD88), cell chemotaxis (CCL-2, -8, -5, 18, 19, 21, CXCL-9, -10, -12, -14, CXCR-2, -3, -6), cell migration (PDGFRA, JAMA, PTPN6, CD274), and angiogenesis (VEGF, IL18, CXCR3, TGFβ1, ACVRL1, COL15A1) in AITL. Furthermore, we found DEGs related to immune function, which were over-represented in AITL samples, and under-represented in T samples, such as CD274 (PD-L1), PDCD1LG2 (PD-L2), and multiple TLRs (TLR1, TLR2, TLR4, TLR8, TLR9, TLR10) (Fig.1B). Those may be related to the development, tumor microenvironment and treatment sensitivity of AITL. KEGG functional enrichment analysis suggested that DEGs in AITL samples were mainly enriched in the ECM-receptor interaction, cytokine-cytokine receptor interaction, PI3K-Akt signaling pathway, NF- κB signaling pathway, cell cycle, apoptosis, and TNF signaling pathway (Fig. 1D). To further explore the relationship between protein and protein, we constructed a PPI network of 25 DEGs based on GO and KEGG pathway analyses (Supplementary document 1), including the most significant central genes in the network: TLR2, TLR4, and CXCL9. Furthermore, we found that PD-L1 had complex interactions with TLR9 in the network (combined score: 0.449; Fig. 1E; Table 3).

### Clinical characteristics

The clinical characteristics of the 88 patients diagnosed with AITL are summarized in Table 1. The patients included 56 males (64%) and 32 females (36%), with a median age of 58 years (range, 17-81 years). Among the patients, systemic B symptoms were described in 53 patients (60%), and 88% of patients had advanced-stage (III and IV) disease by Ann Arbor classification. Almost all the patients had more than one extranodal site, and bone marrow (38%) was the most commonly involved extranodal site, followed by spleen (36%), skin (22%), and liver (13%). Autoimmune hemolytic anemia was observed in 39% of patients. Most AITL patients show elevated serum lactate dehydrogenase (LDH) levels, elevated serum β_2_-microglobulin (β_2_-MG) levels, and elevated C-reactive protein levels (84%, 66%, and 33%, respectively). Data on autoantibodies, cryoglobulinemia, and circulating immune complexes were not available for all patients, but antinuclear antibody was found in 33% of patients. Patients were stratified by IPI score; a total of 60% of patients had high-intermediate or high risk. Additionally, Epstein-Barr virus (EBV)-positive status was detected by quantitative PCR in 20 of 25 (80%) patients.

### Pathological characteristics

**Figure 2:**
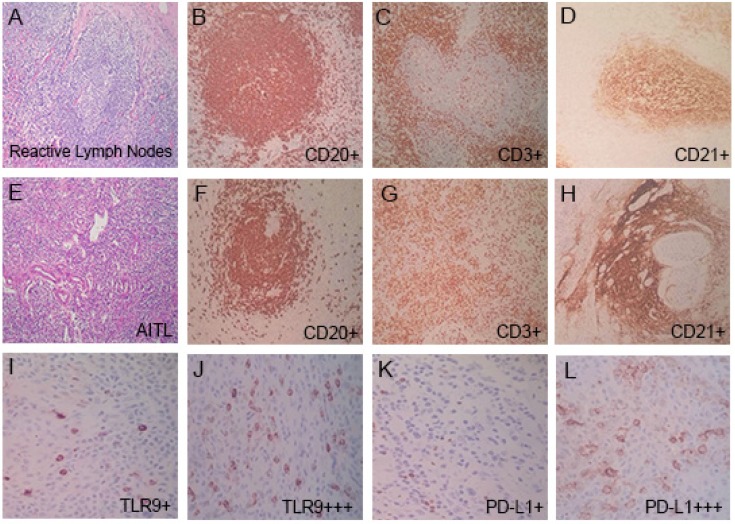
Pathologic features in reactive lymph nodes and AITL tissues. **(A-D)** In reactive lymph nodes, the presence of numerous CD20^+^ B cells **(B)**, CD3^+^ T cells **(C)**, and CD21^+^ FDC **(D)**. **(E-L)** In AITL tissues, nodal biopsy exhibited vascular proliferation and numerous intermediate-sized lymphoid cells. The presence of rare CD20^+^ B cells **(F)**, numerous CD3^+^ T cells **(G)**, and an expanded CD21^+^ FDC meshwork in AITL tissues **(H)**. TLR9 low and high expression **(I, J)**, and PD-L1 low and high expression **(K, L)** in AITL tissues.

Reactive lymph node samples displayed follicular hyperplasia, the germinal centers were mainly composed of CD20-positive B cells, and the interfollicular areas were mainly composed of CD3-positive T cells (Fig. 2A-2D). In AITL, nodal biopsy exhibited vascular proliferation and numerous intermediate-sized lymphoid cells with irregular nuclei and clear cytoplasm (Fig. 2E-2H). The presence of numerous CD3-positive T cells, CD20 highlighted rare B cells, and an expanded CD21-positive follicular dendritic cell (FDC) meshwork associated with neoplastic T cell proliferation was shown, which is a characteristic feature of AITL. Immunohistochemical examination of tumor cells showed CD3 was positive in 98% (86/88) of patients, CD4 was positive in 92% (81/88), CD10 in 57% (50/88), CD30 in 80% (70/88), BCL-6 in 56% (49/88), and CXCL13 in 88% (77/88) in patients with AITL, and the high expression rates of Ki67 (≥ 45%) were 59% (52/88) (Table 2 ).

### Treatment and outcome

**Figure 3:**
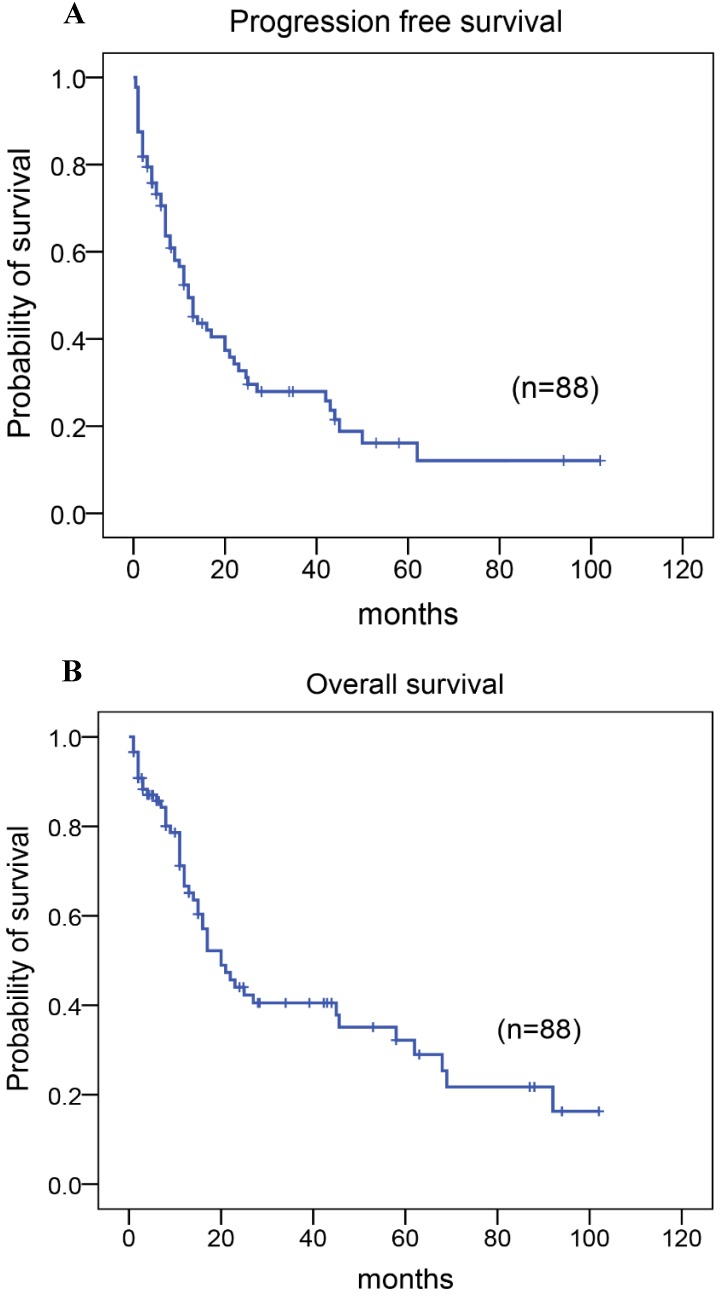
Kaplan-Meier analysis for PFS and OS in patients with AITL. **(A)** PFS. **(B)** OS.

Among the 88 patients, 79 patients received chemotherapy with CHOP, CHOPE, or Hyper CVAD regimen, including five patients who received adjuvant radiotherapy following chemotherapy as the first-line treatment, two patients who did not receive any therapy, and seven patients who had autologous stem cell transplantation as consolidation therapy after achieving the first complete response/remission (CR) (Table 1). A total of 28 patients (32%) achieved CR or unconfirmed CR. The 5-year progression-free survival (PFS) rate was 22%, the median time of PFS was 12.00 months (95% CI: 8.539-15.461; Fig. 3A). And the 5-year overall survival (OS) rate for 88 patients with AITL was 37%, the median time of OS was 20.00 months (95% CI:13.558-26.442; Fig. 3B). At the close of the study, 55% of patients had died.

### TLR9 and PD-L1 expression in AITL

The expression of TLR9 and PD-L1 in AITL tissues by immunohistochemical staining were shown (Table 2; Figure 2). Of the 88 patients, 84% (74/88) patients had PD-1 high expression. Immunohistochemistry with PD-L1 showed that 50% (44/88) patients had PD-L1 high expression and the rest (50%) were classified as PD-L1 low expression. 69% (61/88) patients were had TLR9 high expression, and 31% (27/88) patients had TLR9 low expression. We further discussed the relationship between TLR9 and PD-L1 expression. Among PD-L1 high expression group (n=44), 70% (31/44) patients had TLR9 high expression, and 30% (13/44) patients had TLR9 low expression. However, there was no significant correlation between TLR9 and PD-L1 expression in AITL (Spearman 'r = 0.025, *P* = 0.820).

### Expression of PD-L1 and TLR9 correlated with reduced OS in AITL

The correlations between TLR9 expression and clinicopathological features were analyzed in this study (Table 4). Compared to the TLR9 low expression group, TLR9 expression was significantly associated with age (*P*=0.006), gender (*P*=0.013), ECOG score (*P*=0.044), and serum β_2_-MG levels (*P*=0.019). In addition, the relationships between PD-L1 expression and clinicopathological features were also summarized in Table 4. We found that PD-L1 expression was associated with B symptoms (*P*=0.050). No statistical associations were observed between PD-L1 expression and age, gender, ECOG score, IPI score, LDH levels, leukocyte count, or Ki-67 expression.

**Figure 4:**
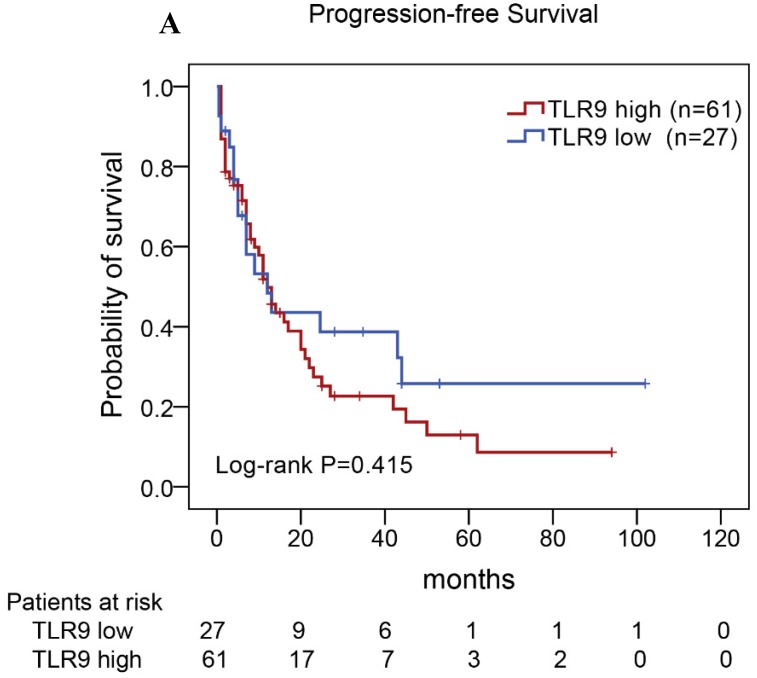

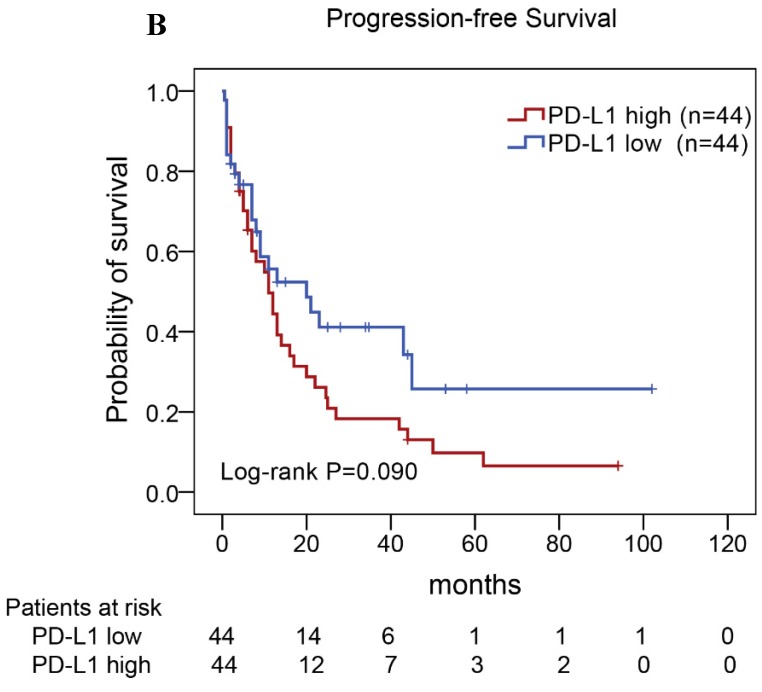

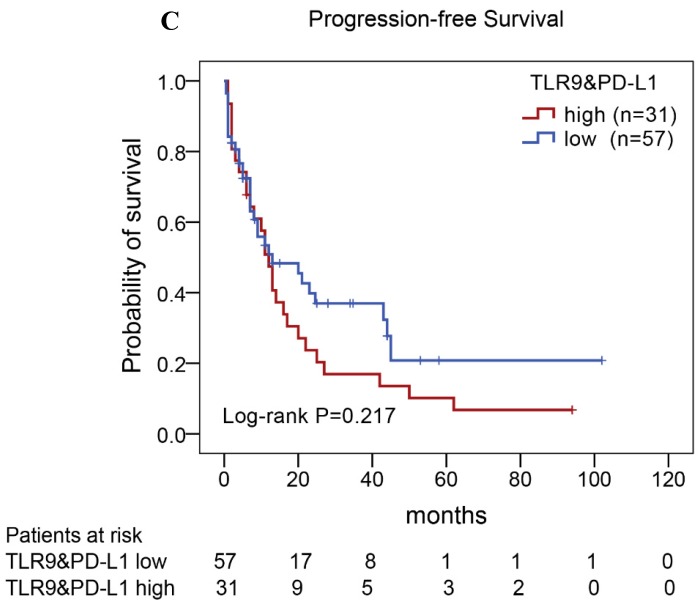

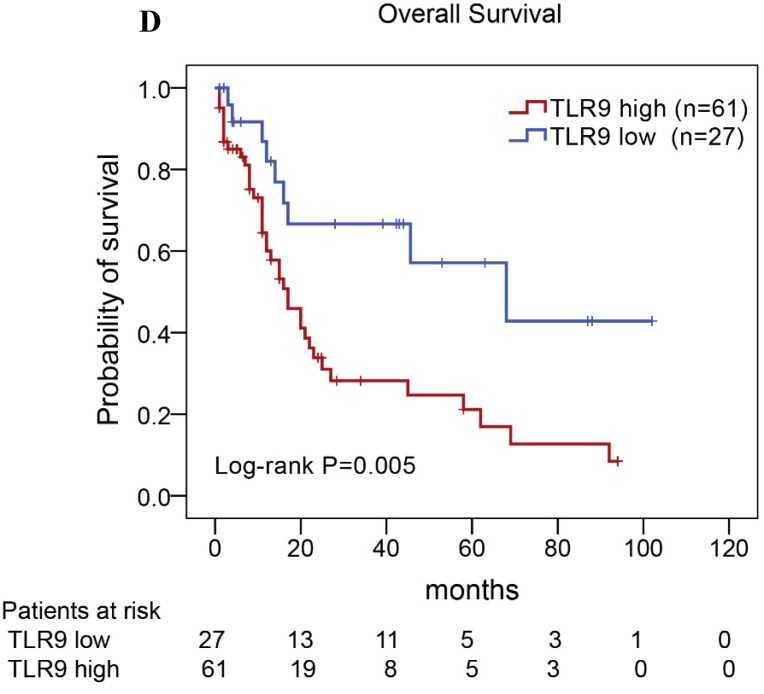

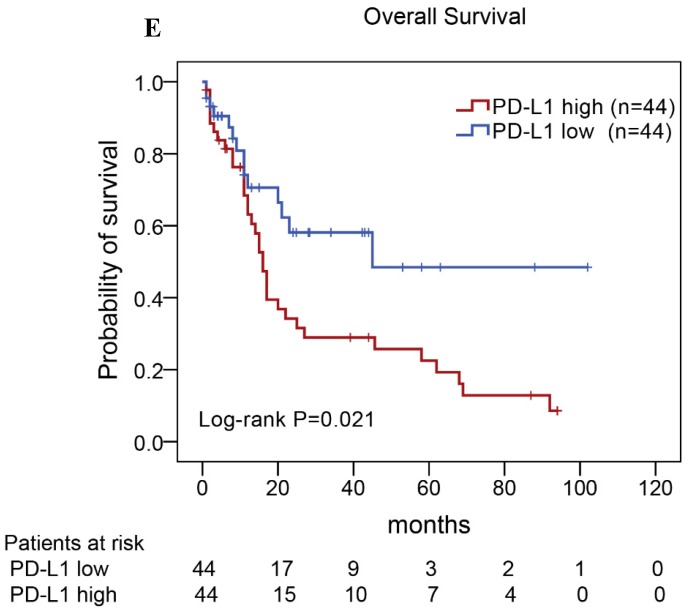

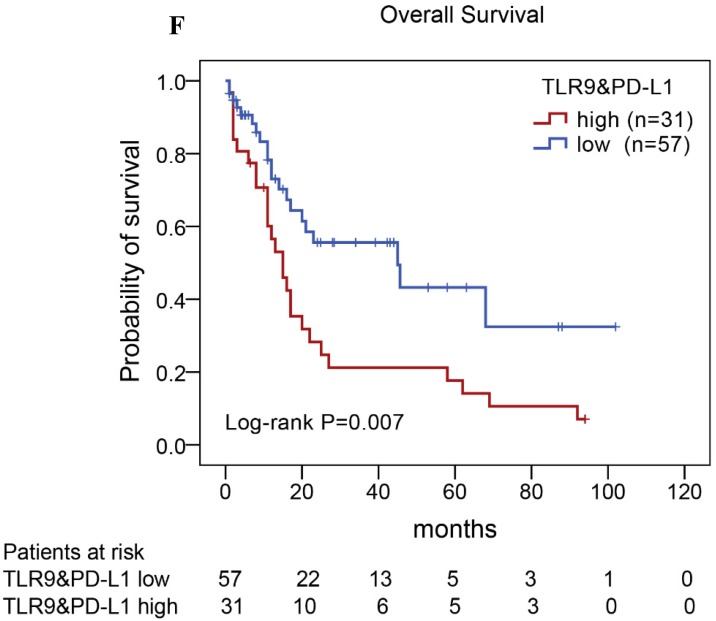
Kaplan-Meier analysis for PFS and OS according to TLR9 and PD-L1 expression in patients with AITL. **(A-C)** Kaplan-Meier analysis for PFS according to TLR9 expression **(A)**, PD-L1 expression (B), and both TLR9 and PD-L1 expression **(C)** in patients with AITL**. (D-F)** Kaplan-Meier analysis for OS according to TLR9 expression **(D)**, PD-L1 expression **(E)**, and both TLR9 and PD-L1 expression **(F)** in patients with AITL.

Of the 88 patients with AITL, we found that the 3-year PFS rate for AITL patients with TLR9 high expression was 22%, the median time of PFS was 12 months (Fig. 4A). The 3-year PFS rate for AITL patients with PD-L1 high expression was 19%, the median time of PFS was 11 months (Fig. 4B). And the 3-year PFS rate for AITL patients with both high expression of TLR9 and PD-L1 was 17%, the median time of PFS was 12 months, respectively (Fig. 4C). Although some of the* P*-value of comparison between the high expression group and the low expression group for PFS was larger than 0.05, the median PFS time was consistently shorter and the PFS time for patients with high expression group was shorter than that for patients with the low expression group. In addition, we further analyzed the correlations between TLR9 and PD-L1 expression and over survival in patients with AITL. AITL patients with high TLR9 expression had a shorter OS compared with low TLR9 expression (17 vs. 68 months; Log-rank *P*= 0.005; Fig. 4D). AITL patients with high PD-L1 expression had worse OS (16 vs. 45 months; Log-rank *P*=0.021; Fig. 4E) compared with low PD-L1 expression. Furthermore, AITL patients with the high expression of both TLR9 and PD-L1 had the worst overall survival rate than patients with the single-high and double low expression (15 vs. 45 months; Log-rank *P*=0.007; Fig. 4F).

### Univariate and multivariate analyses of prognostic factors in AITL

We performed univariate and multivariate analyses of OS using the Cox proportional hazards model to evaluate the prognostic factors (Table 5). Univariate analysis revealed that TLR9 high expression (Hazard Ratio, HR: 2.677, *P*=0.008), PD-L1 high expression (HR: 2.037, *P*=0.026), PD-1 high expression (HR: 3.078, *P*=0.013), ECOG status ≥2 (HR: 2.672, *P*=0.032), Ann Arbor stage III or IV (HR: 3.404, *P*=0.021), elevated serum LDH levels (HR: 3.442, *P*=0.019), elevated serum β_2_-MG levels (HR: 1.959, *P*=0.036), and high IPI score (HR: 5.570,*P*=0.029) were an individual unfavorable prognostic factors. Multivariate analysis also indicated that TLR9 high expression, PD-L1 high expression, ECOG status ≥ 2, Ann Arbor stage III or IV, elevated serum β_2_-MG levels were poor prognosis factors in patients with AITL.

**Figure 5:**
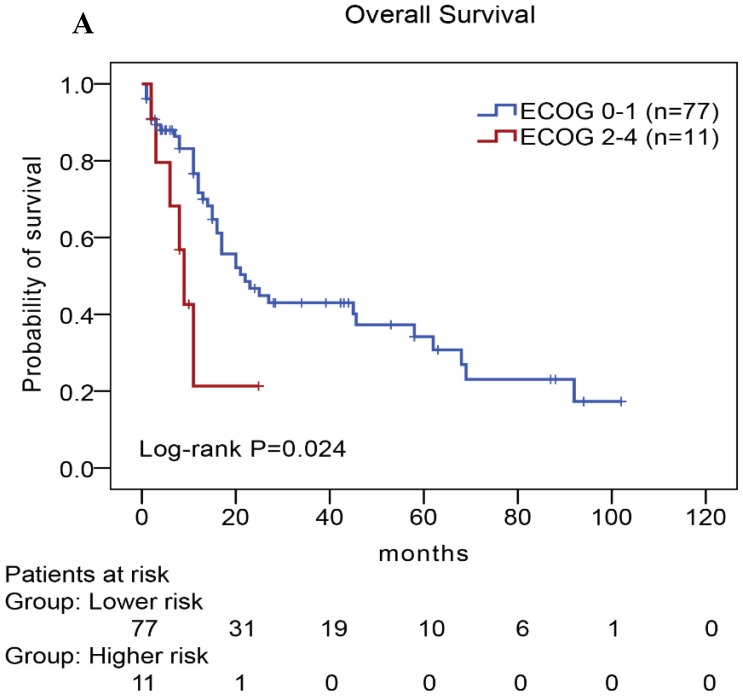

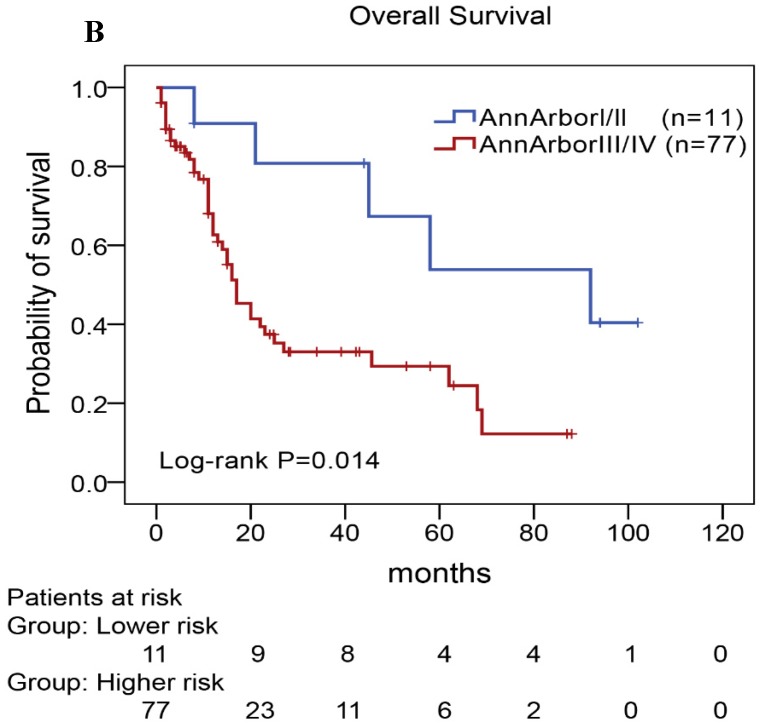

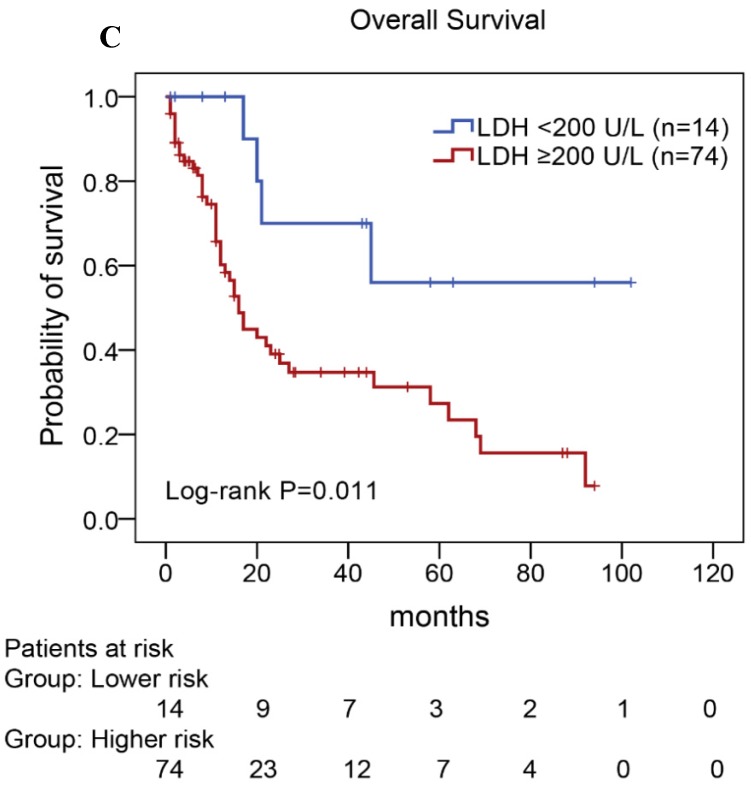

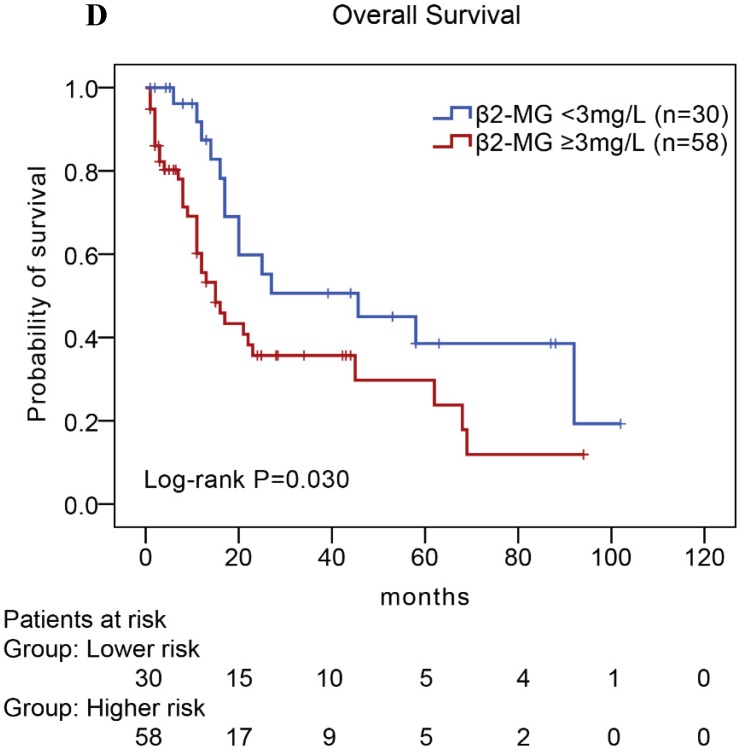

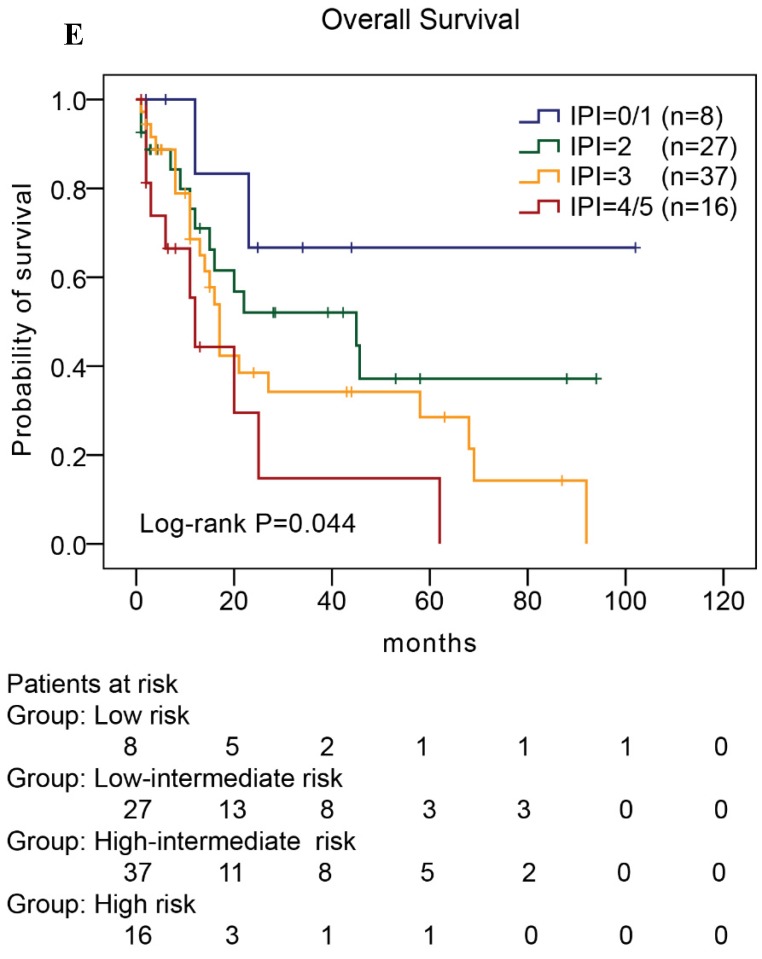
Kaplan-Meier analysis for OS according to ECOG score (A), Ann Arbor stage (B), serum LDH levels (C), serum β_2_-MG levels (D), and IPI score (E) in patients with AITL.

In our study, we further found inferior overall survival in AITL with clinical features of ECOG status ≥ 2, advanced-stage, elevated serum LDH levels, elevated serum β2-MG levels, and high IPI score (Fig. 5). And we further demonstrated that AITL patients with IPI scores of 0 or 1, 2, 3, and 4 or 5 had 2-year OS rates of 68%, 52%, 47%, and 29%, respectively, and high IPI score predicted shorter OS in patients with AITL (Log-rank *P*=0.044; Fig. 5E).

## Discussion

Due to low incidence, the clinical characteristics and prognosis evaluation of AITL remain poorly understood. In this study, we identified DEGs in AITL by bioinformatics analysis, which may help distinguish AITL from other T-cell lymphoma subtypes. Meanwhile, we studied 88 AITL patients who received treatment at Harbin Medical University Cancer Hospital, which is a large study for a single center in China. And we explored TLR9 and PD-L1 expression in AITL tissues by immunohistochemical staining, and evaluated the association between those expression and clinicopathological features, which may disclose clinical significance of TLR9 and PD-L1 expression in AITL.

Recent study with gene expression profiling of 114 AITL reported that the cytokine environment includes angiogenesis, inflammatory, immunosuppressive and many chemokines/receptors, indicating a complex immune network in AITL 17, which are consistent with our results. In our study, we further identified several DEGs related to immune function including PD-L1, PD-L2, and multiple TLRs, which may play a critical role in the progression of AITL. Based on KEGG pathway analysis, we also evaluated several cancer-related pathways in AITL, including PI3K-Akt signaling pathway, NF-κB signaling pathway, cell cycle, apoptosis, and TNF signaling pathway. Previous study reported that the NF-κB pathway, immunosuppressive pathways (TGFβ pathway) and IL-6 signaling were enriched in AITL 18. And Zhou LL et al. suggested that activation of PI3K/AKT, JAK-STAT, and NF-κB signaling pathway are associated with processes of T-cell lymphoma 19. These pathways may play an important role in invasion, metastasis, and therapeutic applications for AITL.

Several studies have demonstrated strong PD-1 expression on tumor-infiltrating lymphocytes in the majority of patients with AITL, and PD-1 expression is the most sensitive marker for AITL 20. In our study, we reported PD-1 and PD-L1 expression in AITL tissue by immunohistochemical staining. However, we found 14 patients with PD-1 low expression, and further found that these patients had CXCL13, CD10, BCL-6 and other Tfh-related antigen expression, meanwhile, combined with pathomorphological features to confirm the diagnosis of AITL, which was consistent with previous reports 20,21. Moreover, we demonstrated that patient with high PD-1 expression had poorer OS compared with low PD-1 expression, and high expression of both PD-1 and PD-L1 had the worst survival rate when compared with the single-high and double low expression. These suggested that the PD-1/PD-L1 expression may contribute to a worse clinical outcome in AITL and may be useful as a clinical prognostic indicator to select appropriate therapeutic approaches for individual patients.

In studies on TLRs and cancer, TLR9 is also expressed in lymphoma cells including oral cancer, breast cancer, glioma and pancreatic cancer cells. Several studies reported that the high expression of TLR9 is associated with tumor growth and migration, and it has been recognized as a new prognostic biomarker in cancer. However, the prognostic significance of TLR9 expression in malignancy is still controversial. Previous studies suggested that the high expression of TLR9 was significantly associated with a shorter OS time 22, 23. However, Leppänen et al. suggested that TLR9 high expression indicated a more favorable prognosis 24. In this study, we analyzed the expression and clinical relevance of TLR9 in AITL and demonstrated that TLR9 expression was an independent prognostic factor, indicating poor prognosis, which suggested that abnormal TLR9 levels may potentially influence the progression of AITL.

In this study, we further evaluated the correlation between TLR9 expression and PD-L1 expression in a relatively large population with AITL. We constructed a PPI network by bioinformatic analysis, and intuitively shown that TLR9 expression and PD-L1 expression displayed complex interactions. Furthermore, using immunohistochemical staining, we found a potentially relevant trend between TLR9 expression and PD-L1 expression in patients with AITL. Among PD-L1 high expression group, 70% patients had high TLR9 expression, which the number of patients was significantly higher than that of patients with TLR9 low expression. In addition, recent studies have reported the possible potential association between TLR9 and PD-L1 expression. Some *in vitro* studies demonstrated that TLR9 stimulation can induce PD-L1 expression in mouse tumor cells 25. In lung cancer, Chen et al. suggested that TLR9 activation in combination with irradiation regulated PD-L1 expression via the NF-kB signaling pathway 26. Wang D et al. also reported that TLR9 stimulation increased immune checkpoint gene expression, such as PD-L1, in the murine syngeneic A20 lymphoma 27. Those indicated that PD-L1 expression may be increased or induced by TLR9 ligands. And this may explain that AITL patients with the high expression of both TLR9 and PD-L1 had the worst overall survival rate than patients with the single-high and double low expression in our study. Our found was consistent with previous studies, and indicated the possible potential association between TLR9 and PD-L1 expression. In addition, we will expand the sample size to further confirm our conclusion, and functional studies on the correlation between TLR9 and PD-L1 expression in AITL are still further explored.

In this study, we also analyzed the correlations between TLR9 and PD-L1 expression and survival time in patients with AITL. We found that the median PFS time for patients with low or high expression of those markers was consistently shorter and the PFS time for patients with high expression was shorter than that for patients with the low expression group. Those indicated that AITL patients with high expression had disease progression at an early stage. In addition, the 5-year OS rate for AITL patients with PD-L1 high expression was only 24%, indicating that the disease has strong clinical aggressive characteristics, which was consistent with the characteristics of AITL reported in the previous literature. Thus, exploring the prognostic factors in AITL remain an area of clinical interest. We reported that IPI scores were significant factors in predicting the survival of patients with AITL. We further reported that ECOG performance status, Ann Arbor stage III or IV, elevated serum LDH levels, and elevated serum β_2_-MG levels were prognostic factors for a shorter OS. However, the application of IPI and PIT scores for AITL is still controversial. Some studies suggested that IPI is not a good prognostic model for AITL 28, while others have supported the prognostic value of IPI in AITL 29,30. In addition, factors not used in IPI, such as IgA, anemia, bone marrow involvement, also significantly affected prognosis 30. Additionally, study has suggested that circulating EBV DNA levels were a prognostic factor for survival 31. A large population and multicenter study is needed to confirm the findings.

In conclusion, we demonstrated that TLR9 and PD-L1 expression indicates a poor prognosis in patients with AITL. The identification of specific prognostic factors for patients with AITL may improve our ability to identify alternative therapies. With the development of novel and complex technologies in genomics and proteomics, significant advances in AITL treatment can be expected in the future.

## Figures and Tables

**Table 3:** The correlation between PD-L1 expression and TLR9 expression on the protein-protein interaction network

Protein 1 expression	Protein 2 expression	Combined score*
PD-L1	TLR9	0.449

* The combined score of >0.4 was considered to be statistically significant using Cytoscape.

**Table 1:** Clinical characteristics at diagnosis of 88 patients with AITL

Characteristic	No. of Patients	
	(n/N)	%
**Age, year, median (rang)**	58(17-81)	
< 60 y	46/88	52
≥ 60 y	42/88	48
**Sex**		
Male	56/88	64
Female	32/88	36
**ECOG score**		
0-1	77/88	88
2-4	11/88	12
**B-symptom**		
Absent	35/88	40
Present	53/88	60
**Tumor Size**		
< 7.5cm	84/88	95
≥ 7.5cm	4/88	5
**Primary site**		
Lymph node	83/88	94
Extranodal	5/88	6
**Ann Arbor stage**		
I or II	11/88	12
III or IV	77/88	88
**Extranodal sites > 1**	**88/88**	**100**
**Mediastinal lymphadenopathy**	**45/88**	**51**
**Extranodal involvement**		
Liver	11/88	13
Splen	32/88	36
Lung	5/88	6
Skin	19/88	22
Kidney	1/88	1
Breast	1/88	1
Gastrointestinal tract	1/88	1
**BM involvement**	33/88	38
**WBC count > 10×10^9^/L**	27/88	31
**Neutrophil count > 8×10^9^/L**	25/88	28
**Lymphocyte count < 0.7×10^9^/L**	25/88	28
**Platelet counts < 100×10^9^/L**	21/88	24
**Anemia (HGB < 120 g/L)**	34/88	39
**Elevated LDH ( > 200 U/L)**	74/88	84
**Elevated β_2_-MG (> 3 mg/L)**	58/88	66
**Elevated CRP (> 20mg/L)**	14/42	33
**Coombs test (positive)**	**3/9**	**-**
**ANA (positive)**	**4/12**	**-**
**EBV DNA (positive)**	**20/25**	**-**
**IPI score**		
0 or 1 (low risk)	8/88	9
2 (low-intermediate risk)	27/88	31
3 (high-intermediate risk)	37/88	42
4 or 5 (high risk)	16/88	18
**Treatment**		
Chemotherapy	79/88	90
Adjuvant radiation	5/79	-
Transplantation	7/88	8
No chemotherapy or radiation	2/88	2
**CR or CR (u)**	28/88	32

Abbreviations: ECOG, Eastern Cooperative Oncology Group; BM, bone marrow; WBC, white blood cell; HGB, Hemoglobin count; LDH, lactate dehydrogenase; β_2_-MG, β_2_-microglobulin; CRP, C-reactive protein; ANA, antinuclear antibody; EBV, Epstein-Barr virus; IPI, international prognostic index; CR complete response/remission.

**Table 2:** Pathologic features in 88 patients with AITL

Immunohistochemistry	NO.(n)	%
CD3	86	98
CD4	81	92
CD5	63	72
CD20	69	78
CD30	70	80
CD21	80	91
CD10	50	57
CXCL13	77	88
PD-1	74	84
BCL-6	49	56
MUM-1	46	52
CD43	13	15
CD79a	8	9
PD-L1	44	50
TLR9	61	69
Ki67 (≥ 45%)	52	59

**Table 4:** Correlations between TLR9 and PD-L1 expression and clinicopathological features in patients with AITL

Characteristics	TLR9		*P-*value	PD-L1		*P*-value
	Low (n=27)	High (n=61)		Low (n=44)	High (n=44)	
Age(years)						
< 60	20	26	0.006	21	25	0.393
≥ 60	7	35		23	19	
Gender						
Male	12	44	0.013	30	26	0.375
Female	15	17		14	18	
Primary site						
Nodal	24	59	0.335	42	41	1.000
Extra nodal	3	2		2	3	
Tumor size						
< 7.5cm	27	57	0.420	40	44	0.125
≥ 7.5cm	0	4		4	0	
Ann Arbor stage						
I-II	3	8	1.000	5	6	0.747
III-IV	24	53		39	38	
B symptoms						
Absent	11	24	0.902	13	22	0.050
Present	16	37		31	22	
ECOG score						
0-1	27	50	0.044	37	40	0.334
2-4	0	11		7	4	
IPI score						
Low risk	4	4	0.097	7	1	0.141
low-intermediate risk	9	18		14	13	
high-intermediat risk	13	24		16	21	
High risk	1	15		7	9	
LDH level (U/L)						
< 200	6	8	0.447	9	5	0.244
≥ 200	21	53		35	39	
HGB level (g/L)						
< 120	8	26	0.248	17	17	1.000
≥ 120	19	35		27	27	
Platelet count(10^9/L)						
< 100	6	15	0.810	14	7	0.080
≥ 100 Leukocyte count(10^9/L)	21	46		30	37	
< 7.2	15	35	0.874	24	26	0.667
≥ 7.2	12	26		20	18	
Lymphocyte count(10^9/L)						
< 0.7	8	17	0.866	11	14	0.478
≥ 0.7	19	44		33	30	
β_2_-MG level						
< 3.0	14	16	0.019	13	17	0.368
≥ 3.0	13	45		31	27	
Ki-67						
< 45	19	33	0.152	22	30	0.083
≥ 45	8	28		22	14	

Abbreviations: ECOG, Eastern Cooperative Oncology Group; IPI, international prognostic index; LDH, lactate dehydrogenase; HGB, Hemoglobin; β_2_-MG, β_2_-microglobulin.

**Table 5:** Prognostic factors affecting the overall survival of patients with AITL

Characteristics	Univariate analysis			Multivariate analysis 1*			Multivariate analysis 2**		
	HR	(95%CI)	*P*-value	HR	(95%CI)	*P*-value	HR	(95%CI)	*P*-value
TLR9: High vs. Low	2.677	(1.291-3.552)	0.008	2.526	(1.157-5.514)	0.020			
PD-L1: High vs. Low	2.037	(1.089-3.811)	0.026				2.620	(1.344-5.107)	0.005
PD-1: High vs. Low	3.078	(1.271-7.451)	0.013						
Age (years): ≥ 60 vs. < 60	1.450	(0.818-2.570)	0.203						
Gender: Male vs. female	1.410	(0.762-2.606)	0.274						
Primary site: Extranodal vs. Nodal	0.430	(0.059-3.134)	0.405						
Tumour size: ≥ 7.5 cm vs. < 7.5 cm	0.871	(0.119-6.352)	0.892						
Ann Arbor stage: III-IV vs. I-II	3.404	(1.202-9.640)	0.021	3.793	(1.309-10.986)	0.014	3.347	(1.169-9.579)	0.024
B symptoms: Present vs. Absent	1.601	(0.866-2.961)	0.134						
ECOG score: 2-4 vs. 0-1	2.672	(1.087-6.569)	0.032	1.673	(0.676-4.143)	0.266	3.063	(1.184-7.925)	0.021
IPI score									
4/5 vs. 0/1	2.188	(0.493-9.709)	0.303						
4/5 vs. 2	3.444	(0.810-14.639)	0.094						
4/5 vs. 3	5.570	(1.188-26.115)	0.029						
LDH levels (U/L): ≥ 200 vs. < 200	3.442	(1.229-9.635)	0.019						
HGB levels (g/L): < 120 vs. ≥ 120	1.083	(0.597-1.962)	0.793						
Platelet count (10^9/L): ≥ 150 vs. < 150	1.179	(0.550-2.526)	0.673						
Leukocyte count(10^9/L): < 7.20 vs. ≥ 7.20	0.895	(0.498-1.611)	0.712						
Lymphocyte count(10^9/L): < 0.7 vs. ≥ 0.7	1.634	(0.850-3.140)	0.141						
β_2_-MG level (mg/L): ≥ 3.0 vs. < 3.0	1.959	(1.046-3.670)	0.036	1.455	(0.755-2.804)	0.263	2.004	(1.068-3.762)	0.030
Ki-67: ≥ 45 vs. < 45	0.550	(0.296-1.024)	0.059						

Abbreviations: HR, hazard ratio; CI, confidence interval; ECOG, Eastern Cooperative Oncology Group; IPI, international prognostic index; LDH, lactate dehydrogenase; HGB, Hemoglobin count; β2-MG, β2-microglobulin. *The variables included in multivariate analysis 1 for OS were TLR9 expression, Ann Arbor stage, ECOG score and β2-MG level. **The variables included in multivariate analysis 2 for OS were PD-L1 expression, Ann Arbor stage, ECOG score and β2-MG level.
